# *Mycobacterium phlei* cell wall complex directly induces apoptosis in human bladder cancer cells

**DOI:** 10.1038/sj.bjc.6690038

**Published:** 1999-01

**Authors:** M C Filion, P Lépicier, A Morales, N C Phillips

**Affiliations:** Faculté de Pharmacie, Université de Montréal, Montréal, Québec H3C 3J7, Canada; Bioniche, London, Ontario, N6M 1A3, Canada; Department of Urology, Queen's University, Kingston General Hospital, Kingston, Ontario, K7L 2V7, Canada

**Keywords:** apoptosis, bladder cancer cells, mycobacterial cell wall, granulocyte–macrophage colony-stimulating factor interleukin 6, interleukin 12

## Abstract

Intact mycobacteria and mycobacterial cell wall extracts have been shown to inhibit the growth of human and murine bladder cancer. Their mechanism of action is, however, poorly understood. *Mycobacterium phlei* mycobacterial cell complex (MCC) is a cell wall preparation that has mycobacterial DNA in the form of short oligonucleotides complexed on the cell wall surface. In this study, we have investigated the possibility that MCC has anti-cancer activity that is mediated by two different mechanisms – a direct effect on cancer cell proliferation and viability and an indirect effect mediated by the production of interleukin 12 (IL-12), a cytokine known to possess anti-cancer activity. We have found that, although MCC is a potent inducer of IL-12 and IL-6 synthesis in monocytes and macrophages either in vitro or in vivo, it is unable to induce the synthesis of either IL-12, IL-6 or granulocyte–macrophage colony-stimulating factor (GM-CSF) by the human transitional bladder cancer cell lines HT-1197 and HT-1376. MCC is not directly cytotoxic towards these cancer cells, but induces apoptosis as determined by nuclear DNA fragmentation and by the release of nuclear mitotic apparatus protein. *Mycobacterium phlei* DNA associated with MCC is responsible for the induction of apoptosis. Our results indicate that MCC directly effects bladder cancer cells by inhibiting cellular proliferation through the induction of apoptosis, and has the potential for an indirect anti-cancer activity by stimulating cancer-infiltrating monocytes/macrophages to synthesize IL-12. © 1999 Cancer Research Campaign


*British Journal of Cancer* (1999) **79**(2), 229–235
© 1999 Cancer Research Campaign

### *Mycobacterium phlei* cell wall complex directly induces apoptosis in human bladder cancer cells

**MC Filion[1,2], P Lépicier[1], A Morales[3] and NC Phillips[1,2]**

[1]Faculté de Pharmacie, Université de Montréal, Montréal, Québec H3C 3J7, Canada; [2] Bioniche, London, Ontario N6M 1A3, Canada; [3]Department of Urology, Queen's University, Kingston General Hospital, Kingston, Ontario K7L 2V7, Canada

**Summary** Intact mycobacteria and mycobacterial cell wall extracts have been shown to inhibit the growth of human and murine bladder cancer. Their mechanism of action is, however, poorly understood. *Mycobacterium phlei* mycobacterial cell complex (MCC) is a cell wall preparation that has mycobacterial DNA in the form of short oligonucleotides complexed on the cell wall surface. In this study, we have investigated the possibility that MCC has anti-cancer activity that is mediated by two different mechanisms – a direct effect on cancer cell proliferation and viability and an indirect effect mediated by the production of interleukin 12 (IL-12), a cytokine known to possess anti-cancer activity. We have found that, although MCC is a potent inducer of IL-12 and IL-6 synthesis in monocytes and macrophages either in vitro or in vivo, it is unable to induce the synthesis of either IL-12, IL-6 or granulocyte–macrophage colony-stimulating factor (GM-CSF) by the human transitional bladder cancer cell lines HT-1197 and HT-1376. MCC is not directly cytotoxic towards these cancer cells, but induces apoptosis as determined by nuclear DNA fragmentation and by the release of nuclear mitotic apparatus protein. *Mycobacterium phlei* DNA associated with MCC is responsible for the induction of apoptosis. Our results indicate that MCC directly effects bladder cancer cells by inhibiting cellular proliferation through the induction of apoptosis, and has the potential for an indirect anti-cancer activity by stimulating cancer-infiltrating monocytes/macrophages to synthesize IL-12.

**Keywords**: apoptosis; bladder cancer cells; mycobacterial cell wall; granulocyte–macrophage colony-stimulating factor interleukin 6; interleukin 12

Intravesical treatment of superficial bladder cancer with live bacillus Calmette–Guérin (BCG) is an effective anti-cancer immunotherapy (Morales, 1984). However, the use of live BCG organism has been associated with serious undesirable side-effects such as fever, serum sickness-like syndromes, granulomatous infection, sepsis and even death (Lamm et al, 1992). Variability in viability and immunogenicity are other disadvantages associated with the use of live bacteria as immunotherapeutic agents (Lagranderie et al, 1996; Zlotta et al, 1997*a*, 1997*b*), and this has led to the development of other less toxic but effective biological response modifiers. A non-viable mycobacterial cell wall extract (MCWE, US Patent No. 4 744 984) derived from *Mycobacterium phlei* (*M. phlei*) has been shown to have anti-cancer activity. Intravesical therapy with MCWE formulated as a mineral oil emulsion (Regression®, Bioniche, London, Ontario, Canada) has been shown to reduce cancer burden in orthotopic and heterotopic murine bladder cancers (Kadhim et al, 1993; Chin et al, 1996) and in patients with carcinoma in situ of the bladder (Morales and Chin, 1997).

MCWE is derived from *M. phlei*, a Gram-positive micro-organism that is not a recognized pathogen for fish, amphibia, birds and mammals. This ubiquitous mycobacteria can be found in soil, on plants and in drinking water (Mallick et al, 1985; Papapetropoulou et al, 1997). MCWE is a partially delipidated and deproteinized macromolecular complex composed primarily of carbohydrates, peptides and lipids. These molecules form the basis of the two major components of the cell wall, peptidoglycan and glycolipid (Chin et al, 1996). Although the mechanism of action of MCWE against bladder cancer is unknown, *M. phlei* has been reported to be a potent immunostimulator active against viral and parasitic infections (Mallick et al, 1985; Tewari et al, 1996).

*M. phlei* mycobacterial cell complex (MCC) is a cell wall composition wherein the mycobacterial DNA is preserved and complexed on the cell wall surface. We have found that MCC preparations contain approximately 5–10% of *M. phlei*-derived DNA, primarily in the form of short oligonucleotides (Filion et al, 1997). We have found that MCC has the ability to induce in vitro and in vivo the synthesis of a number of human and mouse cytokines (Filion et al, 1998). Because of the reported anti-cancer activity of interleukin 12 (IL-12) towards some cancer cell lines (Izquierdo et al, 1996; Stine et al, 1996) and the stimulatory activity of granulocyte–macrophage colony-stimulating factor (GM-CSF) on bladder cancer cell proliferation (Hawkyard et al, 1993), we felt it appropriate to determine whether MCC could induce the synthesis of these anti- and procancer cytokines by bladder cancer cells. We have also determined whether MCC could induce IL-6, which has been shown to function either as a growth factor (Okamato et al, 1997) or as an antiproliferative factor for bladder cancer lines (Alexandroff et al, 1997).

In this report we have (a) evaluated the ability of MCC to induce the synthesis of IL-6, IL-12 and GM-CSF by human HT-1197 and HT-1376 bladder cancer cells, and (b) determined whether MCC possesses direct cytostatic or cytotoxic activity towards these cancer cells. Our results show that MCC does not have the

*Received 16 March 1998*
*Revised 16 June 1998*
*Accepted 16 June 1998*

*Correspondence to:* NC Phillips, Bioniche Inc., 383 Sovereign Road, London, Ontario, Canada N6M 1A3

capacity to induce the synthesis of IL-12, IL-6 or GM-CSF by bladder cancer cells. However, MCC is a potent inducer of IL-12 and IL-6, and a poor inducer of GM-CSF synthesis by monocytes and macrophages. Furthermore, we show that MCC is able to directly inhibit the proliferation of HT-1197 and HT-1376 transitional cell carcinoma. MCC does not appear to be directly toxic towards these cancer cells: it does, however, induce apoptosis. We found that *M. phlei* DNA associated with MCC is responsible for the induction of apoptosis. Our results indicate that MCC may possess a direct action against human bladder cancer cells after intravesical administration by inducing apoptosis, and an indirect action by stimulating cancer-infiltrating monocytes/macrophages to synthesize IL-12.

### MATERIALS AND METHODS

### Cells

HT-1197 and HT-1376 are cancer cell lines developed from anaplastic transitional cell carcinomas of the bladder taken from a 44-year-old Caucasian man (grade 4) and a 58-year-old Caucasian woman (grade 3) respectively (Rasheed et al, 1977). HT-1197 and HT-1376 cells were obtained from the American Type Tissue Culture Collection (ATCC; Rockville, MD, USA) and were grown in modified Eagle medium (MEM) supplemented with non-essential amino acids and vitamins and containing 10% fetal calf serum (FCS) (MEM-FCS; all from Gibco Life Science, Burlington, Ontario, Canada). Human monocytic THP-1 cells and murine monocytic RAW 264.7 cells obtained from the ATCC were cultured in RPMI-1640 medium supplemented with 10% FCS, 2 mM L-glutamine and 20 mM Hepes (all from Gibco Life Science). Murine macrophages were obtained from female CD1 mice injected i.p. with 1.5 ml sterile Brewer's thioglycolate broth (Difco, Detroit, MI, USA). The peritoneal exudate (> 85% macrophages) was harvested at day 4, washed by centrifugation in Hanks' balanced salt solution (HBSS) and seeded in 6-well flat bottom microplates at $1.0 \times 10^6$ cells ml$^{-1}$ in the medium described for monocytes. The cells were allowed to adhere for 18 h at 37°C in an atmosphere of 5% carbon dioxide, after which non-adherent cells were removed by gentle washing with warm medium. Murine spleen cells were prepared by gentle teasing through sterile stainless-steel screens. Cell suspensions were layered on Lympholyte-M cell separation media (CedarLane, Hornby, Ontario, Canada) and centrifuged at 2200 r.p.m. for 30 min to remove red blood cells and dead cells. The cells were cultured in the culture medium described above. All cells were cultured at 37°C in an atmosphere of 5% carbon dioxide.

### Cytokine analysis

Cells were seeded at $1 \times 10^6$ cells ml$^{-1}$ in 6-well flat-bottomed tissue culture plates and treated with different concentrations of MCC for 48 h. Human (h) and murine (m) IL-6, IL-12 and GM-CSF synthesis was measured after 48 h by means of commercial enzyme-linked immunosorbent assay (ELISA) (all from BioSource, Camarillo, CA, USA, except murine GM-CSF which was from R&D Systems, Minneapolis, MN, USA). The ELISA sensitivities were: 8 pg ml$^{-1}$ for mIL-6, 2 pg ml$^{-1}$ for mIL-12, 1 pg ml$^{-1}$ for mGM-CSF, 104 fg ml$^{-1}$ for hIL-6, 1 pg ml$^{-1}$ for hIL-12 and 1 pg ml$^{-1}$ for hGM-CSF.

### Proliferation assay

HT-1197 and HT-1376 cells were incubated in 1.0 ml MEM-FCS at $3 \times 10^5$ cells in six-well flat-bottomed tissue culture plates with MCC, *Escherichia coli* 011:B4 lipopolysaccharide (LPS; Sigma-Aldrich Canada, Oakville, Ontario, Canada) or recombinant hIL-12 (R&D Systems) for 20 h at 37°C in an atmosphere of 5% carbon dioxide. Cellular growth was measured by determination of dimethylthiazoldiphenyltetrazolium bromide (MTT) reduction (Mosman et al, 1983). Briefly, 100 μl of MTT (Sigma-Aldrich) dissolved in PBS at 5 mg ml$^{-1}$ was added to wells and incubated at 37°C for 4 h. Acid-isopropanol (1.0 ml of 0.04 N hydrochloric acid in isopropanol) was then added, and reduced MTT was measured at a wavelength of 570 nm.

### MCC toxicity determination

HT-1197 or HT-1376 cells were incubated in 1.0 ml of MEM-FCS at $3 \times 10^5$ cells in 6-well flat-bottomed tissue culture plates with MCC for 48 h. Lactate dehydrogenase (LDH) activity in the supernatant was used as an indicator of cell death, and was determined by means of a commercial kit (Sigma-Aldrich). Total LDH activity was determined by incubating macrophages in 1.0% v/v Triton X-100 in water to induce lysis, followed by vigorous agitation (Filion and Phillips, 1997).

### Analysis of apoptosis

HT-1197 or HT-1376 cells were incubated in 1.0 ml MEM-FCS at $3 \times 10^5$ cells in six-well flat-bottomed tissue culture plates with 1 μg ml$^{-1}$ of MCC or with 1 ng ml$^{-1}$ of IL-12 for up to 48 h. After incubation, non-adherent cells in the supernatant were collected by centrifugation at 200 ***g*** for 10 min. The pellet or remaining adherent cells were lysed with 0.5 ml of hypotonic lysing buffer, pH 7.5 (10 mM Tris buffer, 1 mM EDTA, 0.2% Triton X-100). The lysates were centrifuged at 13 000 ***g*** for 10 min and the supernatants containing fragmented DNA were precipitated overnight at –20°C in 50% isopropanol and 0.5 M sodium chloride. The precipitates were collected by centrifugation and analysed by electrophoresis in 0.7% agarose gels for 3 h at 100 V (Newell, 1990). Apoptosis was also determined by an ELISA (Calbiochem, Cambridge, MA, USA) that detects nuclear mitotic apparatus protein (NuMA) released from cells undergoing induced apoptosis (Miller et al, 1993). Briefly, HT-1197 or HT-1376 cells were treated as described above with MCC or with DNA extracted from MCC. Media from the cell cultures was removed at 0, 3, 6, 24 and/or 48 h, centrifuged at 200 ***g*** for 10 min and the supernatant was collected. The supernatant (100 μl) was used for the determination of NuMA protein release by ELISA.

### Isolation and treatment of DNA from MCC

Mycobacterial DNA was purified from MCC by conventional phenol extraction and ethanol precipitation (Moore, 1995). MCC or DNA extracted from MCC was incubated with 1 U of DNAse I (Gibco Life Sciences) for 1 h at 25°C in 20 mM tris-HCl (pH 8.4), 2 mM magnesium chloride and 50 mM potassium chloride. DNAse I was inactivated by the addition of EDTA (final concentration 2.5 mM) and the reaction mixture was heated for 10 min at 65°C. Digestion was confirmed by electrophoresis in 0.7% agarose gels for 3 h at 100 V.

© Cancer Research Campaign 1999

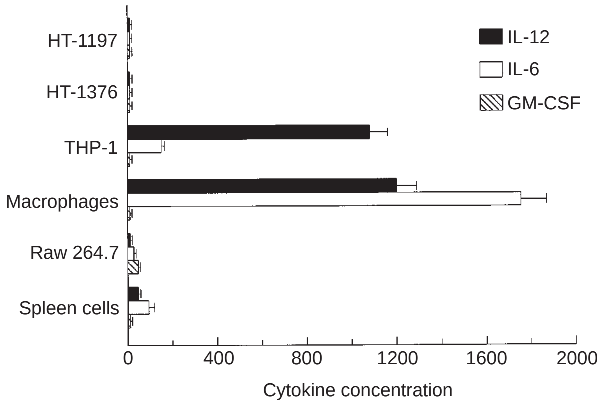

**Figure 1** MCC induces IL-6 and IL-12 synthesis only in monocytes and macrophages. Human bladder cancer cell lines HT-1197 and HT-1376, human monocytic THP-1 cells, murine monocytic RAW 264.7 cells, murine macrophages or murine spleen cells were incubated at 1.0 × 10$^6$ cells ml$^{-1}$ with 1.0 μg ml$^{-1}$ of MCC for 48 h. IL-6, IL-12 and GM-CSF in the supernatant were measured after 48 h by the appropriate ELISA. The results shown are the means ± s.d. of three independent experiments

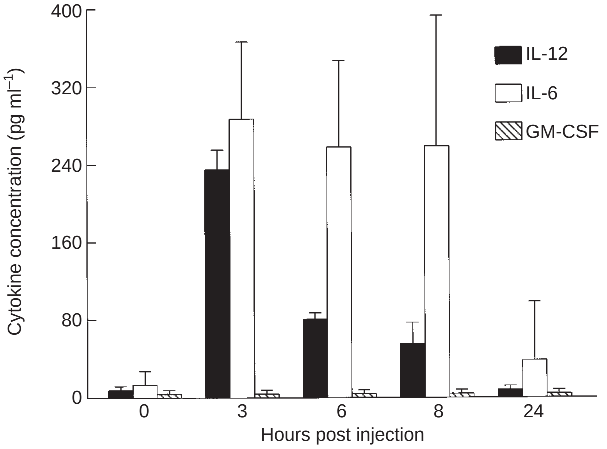

**Figure 2** MCC induces the synthesis of IL-6 and IL-12 without GM-CSF in mice. Groups of five mice were injected i.p. with MCC (50.0 mg kg$^{-1}$). Murine IL-6, IL-12 and GM-CSF in the serum were quantified at 0, 3, 6, 8 and 24 h after injection by the appopriate ELISA. The results shown are the means ± s.d. of two independent experiments

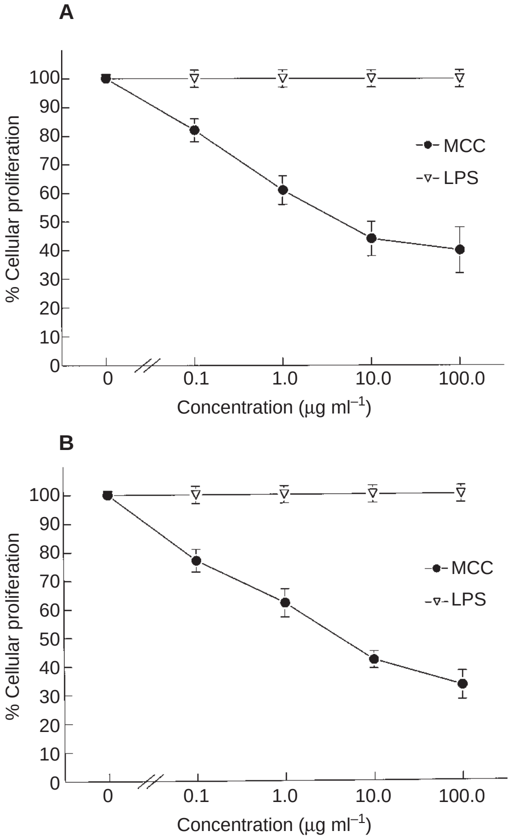

**Figure 3** MCC inhibits the cellular proliferation of human bladder cancer cells. HT-1197 (**A**) and HT-1376 (**B**) cells were incubated at 3.0 × 10$^5$ cells ml$^{-1}$ with different concentrations of MCC or LPS for 24 h at 37°C, 5% carbon dioxide. Cellular proliferation was measured by determination of MTT reduction as described in the Materials and methods section. The results shown are the means ± s.d. of three independent experiments

### RESULTS

### Effect of MCC on cytokine synthesis

A number of cytokines are known to influence the regression or the progression of cancer. IL-12 has been shown to have potent anti-cancer activity by stimulating natural killer (NK) cells or T-cells, and/or in blocking angiogenesis (Voest et al, 1995; Zitvogel and Lotze, 1995; Stern et al, 1996) whereas GM-CSF has the capacity to stimulate the unwanted proliferation of low-grade bladder neoplasms (Hawkyard et al, 1993). IL-6 has the capacity to stimulate the growth of bladder cancer cells (Okamoto et al, 1997) or to inhibit their proliferation (Alexandroff et al, 1997). It has also been shown that bladder cancer cells are able to secrete cytokines (De Reijke et al, 1993; Bevers et al, 1997). We have, therefore, evaluated whether bladder cancer cells or immune effector cells have the ability to synthesize IL-12, IL-6 or GM-C SF in response to MCC. As shown in Figure 1, IL-12, IL-6 and GM-CSF are not produced by HT-1197 and HT-1376 bladder cancer cells in response to 1.0 μg ml$^{-1}$ of MCC. However, at this concentration, human THP-1 monocytes and murine macrophages are able to produce significant amounts of IL-12 and IL-6 in response to MCC treatment. Only murine RAW 264.7 cells were able to synthesize GM-CSF. The i.p. administration of MCC to female CD1 mice did not result in the synthesis of GM-CSF, but did, however, induce the synthesis of IL-12 and IL-6 (Figure 2). Similar results were obtained when MCC was administered i.v. (data not shown).

### Inhibition of cancer cell proliferation by MCC

The ability of MCC to directly influence the proliferation of HT-1197 and HT-1376 bladder cancer cells was investigated. MCC is able to inhibit the cellular proliferation of these bladder cancer cells in a dose-dependent manner, whereas the control immuno-stimulant LPS is without activity (Figure 3). IL-12 has been shown to reduce the proliferative rate of several cancer cell lines (Izquierdo et al, 1996; Stine et al, 1996). IL-12 does not inhibit the proliferation of either HT-1197 or HT-1376 bladder cancer cells in the dose range 0.1–100 ng ml$^{-1}$ (data not shown). To explain this growth inhibition, we have evaluated whether MCC is directly cytotoxic towards these cancer cells. Toxicity is characterized by the loss of plasma membrane integrity and release of the

*© Cancer Research Campaign 1999*

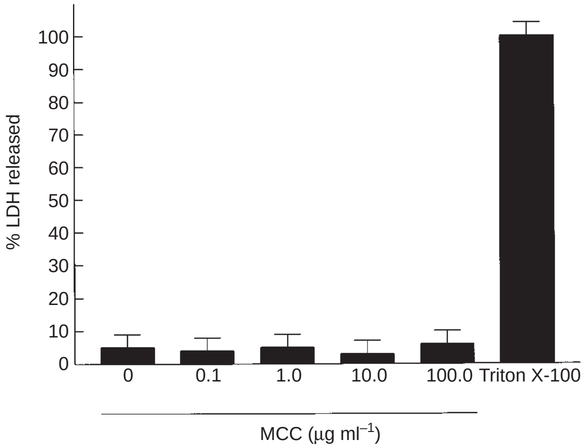

**Figure 4** MCC is not cytotoxic towards human bladder cancer cells. HT-1376 cells were incubated at $3.0 \times 10^5$ cells $ml^{-1}$ with the indicated concentrations of MCC for 48 h at 37°C, 5% carbon dioxide. Toxicity was determined by the release of LDH activity into the supernatant. Total LDH activity was determined by incubating the cells with 1.0% (v/v) Triton X-100. The results shown are the means ± s.d. of three independent experiments cytoplasmic enzyme LDH (Wyllie et al, 1980). The release of LDH into the culture supernatant has been used as an indicator of cell death (Phillips et al, 1996), and human bladder cancer cells have been shown to release LDH when treated with cytotoxic agents (Rahman, 1994). As shown in Figure 4, MCC is not cytotoxic towards HT-1376 cells, as determined by the release of LDH into the culture supernatant. Similar results were obtained with HT-1197 cells (data not shown).

### Induction of apoptosis by MCC

The lack of a direct cytotoxicity of MCC towards HT-1197 and HT-1376 cancer cells in combination with the antiproliferative activity suggested that MCC is an apoptosis inducer. Apoptosis, or programmed cell death, is associated with nuclear DNA fragmentation, the release of nuclear matrix proteins such as NuMA and the loss of cell–substrate contact (Wyllie et al, 1980; Newell et al, 1990; Miller et al, 1993). The induction of apoptosis was demonstrated by visualization of nucleosome-sized DNA fragments on agarose gel electrophoresis and by the release of NuMA proteins. MCC at 1.0 µg $ml^{-1}$ was able to induce apoptosis in both HT-1197 and HT-1376 bladder cancer cells. Nucleosome-sized DNA fragments characteristic of apoptosis were detected in detached HT-1197 and HT-1376 bladder cancer cells after treatment with MCC (Figure 5) MCC also induced the release of NuMA proteins in a dose-related manner (Figure 6A). The release of NuMA increased gradually from 3 h to 48 h; a concentration of 100 µg $ml^{-1}$ MCC being the most effective (Figure 6B and C). IL-12, which has been shown to induce apoptosis of several cancer cell lines (Stine et al, 1996), was unable to induce apoptosis in HT-1197 and HT-1376 bladder cancer cells, as demonstrated by an absence of nucleosome-sized DNA fragments on agarose gel electrophoresis (Figure 5).

We have previously shown that MCC contains approximately 5–10% of *M. phlei*-derived DNA (Filion et al, 1997). We have evaluated whether the DNA associated with MCC can induce apoptosis of bladder cancer cells. DNA extracted from MCC is able to induce the release of NuMA from HT-1376 cells, but to a lesser extent than MCC (Figure 7). Treatment of MCC or *M. phlei* DNA extracted from MCC by DNAse I, which has the capacity to degrade single- and double-stranded DNA, significantly inhibits the release of NuMA proteins (Figure 7), indicating that *M. phlei* DNA plays a pivotal role in the induction of apoptosis in HT-1376 cancer cells. Similar results were obtained with HT-1197 cells (data not shown).

### DISCUSSION

In the present study, we have shown that MCC can act directly on bladder cancer cells by inhibiting cellular proliferation through the

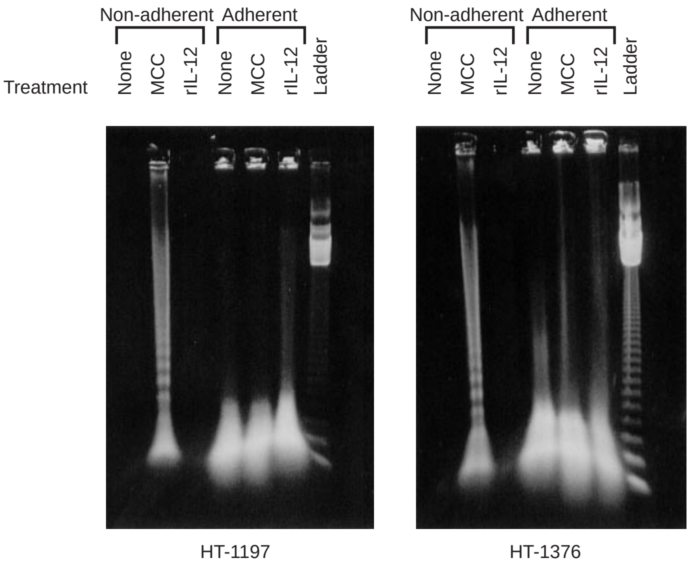

**Figure 5** MCC induces nuclear DNA fragmentation of human bladder cancer cells. HT-1197 and HT-1376 cells were incubated at $3.0 \times 10^5$ cells $ml^{-1}$ with 1.0 µg $ml^{-1}$ of MCC or with 1.0 ng $ml^{-1}$ of IL-12 at 37°C, 5% carbon dioxide. Adherent and non-adherent cells were lysed 48 h later. Cellular DNA was extracted, precipitated and analysed by electrophoresis in 0.7% agarose gels for 3 h at 100 V as described in the Materials and methods section. A 123-bp DNA ladder was used to evaluate the molecular weight of nucleosome-sized DNA. The results shown are for one experiment out of three; all give similar results

 © Cancer Research Campaign 1999

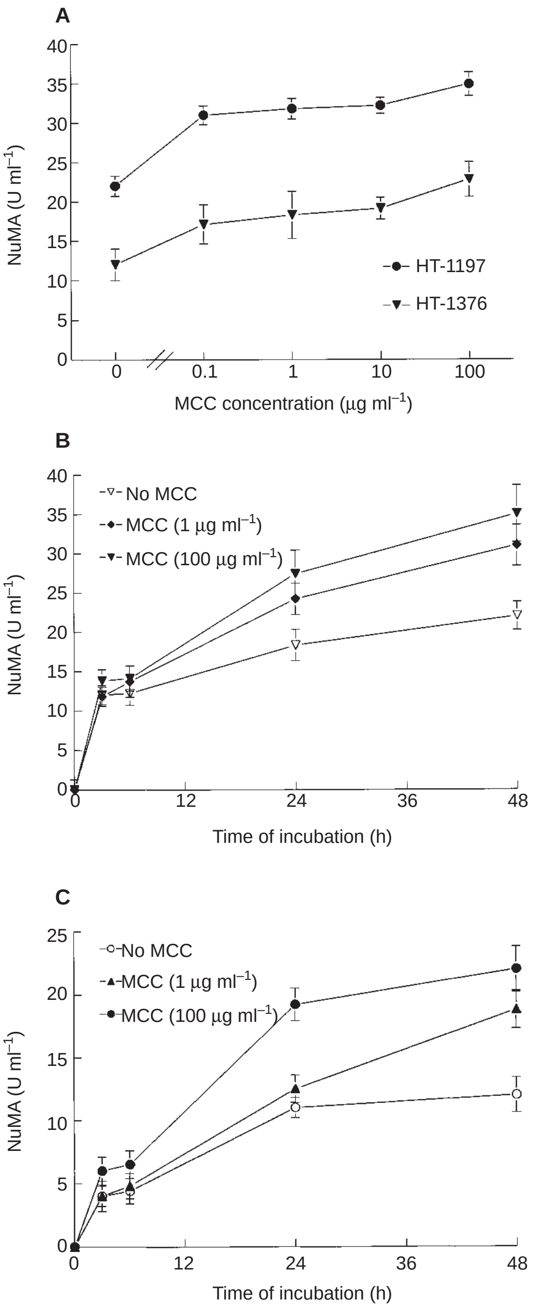

**Figure 6** MCC induces the release of NuMA from human bladder cancer cells. HT-1197 and HT-1376 cells were incubated at $3.0 \times 10^5$ cells $ml^{-1}$ with MCC at 37°C, 5% carbon dioxide. In (**A**), the release of NuMA from the cells into the supernatant was detected by ELISA during incubation with different concentrations of MCC for 48 h. In (**B**), the release of NuMA from HT-1197 cells, and in (**C**) the release of NuMA from HT-1376 cells into the supernatant, was determined at 0, 3, 8, 24 and 48 h after treatment with 1 μg $ml^{-1}$ or 100 μg $ml^{-1}$ of MCC. The results shown are the means ± s.d. of three independent experiments

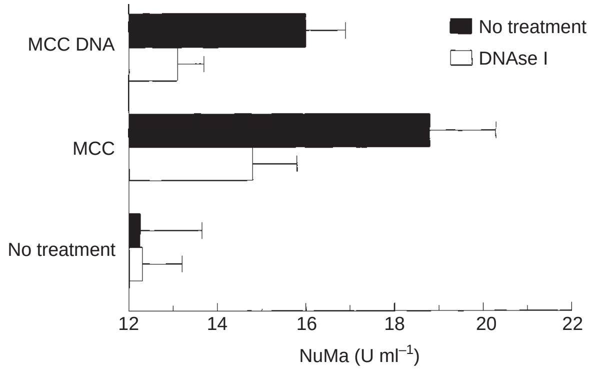

**Figure 7** DNA extracted from MCC induces the release of NuMA. HT-1376 cells ($3.0 \times 10^5$ cells $ml^{-1}$) were incubated for 48 h at 37°C, 5% carbon dioxide with MCC or DNA purified from MCC (1 μg $ml^{-1}$). In some experiments, MCC or DNA purified from MCC were treated by DNAse I (1 U) as described in the Materials and methods section before their addition to HT-1376 cells. After 48 h, supernatants were collected (100 μl) and the release of NuMA released was quantified by ELISA as described in the Materials and methods section. The results shown are the means ± s.d. of two independent experiments induction of apoptosis. MCC does not have the ability to induce the synthesis of anti- (IL-12) or procancer (GM-CSF) cytokines by bladder cancer cells but does have the ability to induce the synthesis of significant amounts of the anti-cancer cytokine IL-12 by monocytes and macrophages either in vitro or in vivo. Monocytes and macrophages have been shown to be present in the bladder wall of healthy individuals and have been shown to accumulate around tumour islands in patients with bladder cancer (El-Demiry et al, 1986; Ioachim-Velogiammi et al, 1994). IL-12 synthesized by these monocytes and macrophages may make a significant contribution to the overall anti-cancer activity of MCC. IL-12 has been shown to possess potent anti-cancer activity after systemic or local administration in mice bearing a variety of malignancies including melanomas, carcinomas, sarcomas, transitional bladder carcinoma and lymphomas (Brunda et al, 1993; Nastala et al, 1994; Noguchi et al, 1995; Zou et al, 1995, Chen et al, 1997). IL-12 plays a role in the development of T-helper type-1 lymphocytes, stimulates γ-interferon (IFN-γ) synthesis, blocks angiogenesis at sites of cancer growth and enhances NK cell and cytotoxic T-cell activity (Voest et al, 1995; Stern et al, 1996; Zitvogel and Lotze, 1996). Although IL-12 can also exert a direct effect on some cancer cells by inhibiting cellular proliferation through the induction of apoptosis (Stine et al, 1996), this does not appear to be the case for the two bladder cancer cell lines used in our study. The addition of recombinant human IL-12 to HT-1197 and HT-1376 bladder cancer cells had no direct effect on cellular proliferation, and apoptosis was not induced by IL-12.

GM-CSF has been shown to induce the proliferation of bladder cancer cells (Hawkyard et al, 1993). The results of our study show that MCC does not induce, either in vitro or in vivo, the synthesis of significant quantities of GM-CSF. BCG possesses the ability to up-regulate the synthesis of IL-6 in bladder cancer cells (Esuvaranathan et al, 1995; Sasaki et al, 1997). The role of IL-6 in bladder cancer outcome is, however, unclear. IL-6 has been shown to function either as a growth factor for bladder cancer lines (Okamoto et al, 1997), or as an antiproliferative factor (Alexandroff et al, 1997). We have found that although MCC at 1.0 μg $ml^{-1}$ does not possess the ability to induce the synthesis of IL-6 by HT-1197 and HT-1376 bladder cancer cells, it does have the ability to induce IL-6 by monocytes and macrophages either in vitro or in vivo.

BCG has been shown to have antiproliferative effects on human bladder cancer cells in vitro in the absence of any immune effector cells (Pryor et al, 1995*a*, 1995*b*). Similar antiproliferative effects of both heat-killed and viable BCG on a panel of human bladder transitional cell carcinoma cell lines have also been described

© Cancer Research Campaign 1999

(Jackson et al, 1994). However, the mechanism of cellular growth inhibition has not been identified in these studies (Jackson et al, 1994; Pryor et al, 1995*a*). Sasaki et al (1997) have shown that the ability of BCG to inhibit the growth of T24 cells was not related to the induction of apoptosis. Other groups have reported an indirect induction of apoptosis in bladder cancer cells by BCG. Shemtov et al (1995) have shown that the human bladder cancer cell line T24 can undergo apoptosis after contact with lymphocyte-activated killer cell (LAK cells), whereas Kudoh et al (1997) have found that BCG-activated lymphocytes are able to induce apotosis of T24 cells via cytokine synthesis. In this study, we have found that the antiproliferative activity of MCC was mediated by the direct induction of apoptosis of human bladder cancer cells and not by a cytotoxic effect. To our knowledge, the present study is the first demonstration of the ability of a mycobacterial cell wall preparation to directly induce apoptosis in cancer cells. We have additionally demonstrated that the apoptosis induced by MCC is related to the presence of mycobacterial DNA in the preparation, and that *M. phlei* DNA has intrinsic apoptosis-inducing activity.

Apoptosis plays an important role in the activity of many anticancer chemotherapeutic agents and in their effectiveness in the treatment of cancer (Dive et al, 1992). It has been shown, however, that bladder carcinomas are resistant to a number of anti-cancer drugs (Lamm et al, 1995). Our data show that MCC not only directly induces apoptosis in HT-1197 bladder cancer cells, which are sensitive to doxorubicin, but also directly induces apoptosis in HT-1376 bladder cancer cells, which have been shown to be resistant to several apoptosis-inducing drugs such as cisplatin and mitomycin C (Kawasaki et al, 1996; Mizutani et al, 1997). Chemotherapy of bladder cancer has been shown to be effective in low-grade cancers, whereas immunotherapy with BCG appears to be optimally effective in higher grade cancers (Lamm, 1995). Although highly effective regardless of tumour grade, BCG treatment is preferably reserved for the most aggressive cancer because of its safety profile (Lamm, 1995). MCC, which appears to possess both a chemotherapeutic-like action against human bladder cancer cells by inducing apoptosis and an indirect BCG-like action by stimulating immune effector cells, has significant promise for the treatment of both low-grade and high-grade bladder cancers. Because mycobacterial cell wall extracts do not contain live bacteria, their safety profile is better (Morales and Chin, 1997). Further studies investigating the role of apoptosis and IL-12 synthesis induced by MCC using in vivo animal models will be required to clarify the mechanisms implicated in the anti-cancer activity of MCC. MCC and *M. phlei* DNA associated with MCC are currently being analysed to determine their proapoptotic activity on a number of other bladder cancer cell lines and on freshly derived primary cultures of human bladder carcinoma cells.

### REFERENCES

Alexandroff AG, Black J, Esuvaranathan K and James K (1997) Antiproliferative effect of IL-6 on transitional cell carcinoma of the bladder. Insight into mechanisms of bacillus Calmette-Guérin in immunotherapy. *Biochem Soc Trans* **25**: 270S

Bevers RF, de Boer EC, Kruth LH and Schamhart DH (1997) Effects of isoniazid on the proliferation and cytokine production of bladder cancer cells in vitro induced by bacille Calmette-Guérin. *Br J Urol* **80**: 35–39

Brunda MJ, Luistro L, Warrier RR, Wright RB, Hubbard BR, Murphy M, Wolf SF and Gately MK (1993) Antitumour and antimetastatic activity of interleukin-12 against murine tumors. *J Exp Med* **178**: 1223–1230

Chen L, Chen D, Block E, O'Donnell M, Kufe DW and Clinton SK (1997) Eradication of murine bladder carcinoma by intratumoral injection of a bicistronic adenoviral vector carrying cDNAs for the IL-12 heterodimer and its inhibition by the IL-12 p40 subunit homodimer. *J Immunol* **159**: 351–359

Chin JL, Kadhim SA, Batislam E, Karlik SJ, Garcia BM, Curtis Nickel J and Morales A (1996) Mycobacterium cell wall: an alternative to intravesical bacillus Calmette Guerin (BCG) therapy in orthotopic murine bladder cancer. *J Urol* **156**: 1189–1193

De Reijke TM, Vos PC, de Boer EC, Bevers RF, de Munick Keizer, Kurth KH and Schambart DH (1993) Cytokine production by the human bladder carcinoma cell line T24 in the presence of bacillus Calmette-Guérin (BCG). *Urol Res* **21**: 349–352

Dive C, Evans CA and Whetton AD (1992) Induction of apoptosis: new targets for cancer chemotherapy. *Semin Cancer Biol* **3**: 417–427

El-Demiry MIM, Hargreave TB, Busuttil A, James K and Chrisholm GD (1986) Immunohistochemical identification of lymphocyte subsets and macrophages in normal human urothelium using monoclonal antibodies. *Br J Urol* **58**: 436–442

Esuvaranathan K, Alexandroff AG, McIntyre M, Jackson AM, Prescott S, Chisholm GD and James K (1995) Interleukin-6 production by bladder tumors is upregulated by BCG immunotherapy. *J Urol* **154**: 572–575

Filion MC and Phillips NC (1997) Toxicity and immunomodulatory activity of liposomal vectors formulated with cationic lipids toward immune effector cells. *Biochim Biophys Acta* **1329**: 345–356

Filion MC, Lépicier P and Phillips NC (1997) *Mycobacterium phlei* cell wall complex, a new antitumoral agent, induces IL-12 synthesis by monocytes/macrophages. *Blood* **90**: 58b

Filion MC et al (1998) (manuscript in preparation)

Hawkyard SJ, Jackson AM, Prescott S, James K and Chisholm GD (1993) The effect of recombinant cytokines on bladder cancer cells in vitro. *J Urol* **150**: 514–518

Ioachim-Velogianni E, Stavropoulos NE, Kitsiou E, Stefanaki S and Agnantis NJ (1994) HLA-DR antigen expression subsets in transitional cell carcinoma of the urinary bladder. An immunohistological study in frozen sections. *J Pathol* **174**: 183–189

Izquierdo MA, Degen D, Sypek JP and Von Hoff DD (1996) Antiproliferative effects of IL-12 on human tumor colony-forming units taken directly from patients. *Anticancer Drugs* **7**: 275–280

Jackson AM, Alexandroff AB, Fleming D, Prescott S, Chisholm GD, Carter GD and James K (1994) Bacillus Calmette-Guérin (BCG) organisms directly alter the growth of bladder tumour cells. *Int J Oncol* **5**: 697–703

Kadhim SA, Chin JL, Batislam E, Karlick S, Garcia BM, Curtis Nickel J and Morales A (1993) Modification of intravesical Bacillus Calmette Guerin (BCG) therapy in orthotopic murine bladder tumor: use of mycobacterial cell wall extract (MCWE). *J Urol* **149**: A225

Kawasaki T, Tomita Y, Bilim V, Takeda M, Takahashi K and Kumanishi T (1996) Abrogation of apoptosis induced by DNA-damaging agents in human bladder-cancer cell lines with *p21*/WAF1/CIP1 and/or *p53* gene alterations. *Int J Cancer* **68**: 501–505

Kudoh S, Liu XX, Mori K and Suzuki T (1997) BCG-activated lymphocytes induce the death of bladder cancer cell via apoptosis in vitro. *Br J Urol* **80S2**: 40

Lagranderie MRR, Balazuc A-M, Deriaud E, Leclerc CD and Gheorghiu M (1996) Comparison of immune responses of mice immunized with five different *Mycobacterium bovis* BCG vaccine strains. *Infect Immunol* **64**: 1–9

Lamm DL (1995) BCG in perspective: advances in the treatment of superficial bladder cancer. *Eur Urol* **27S1**: 2

Lamm DL, van der Meijden AD and Morales A (1992) Incidence and treatment of complications of bacillus Calmette-Guérin intravesical therapy in supperficial bladder cancer. *J Urol* **147**: 596–600

Lamm DL, Riggs DR, Traynelis CL and Nseyo UN (1995) Failure of intravesical chemotherapy prophylaxis in superficial transitional cell carcinoma of the bladder. *J Urol* **153**: 1444–1450

Mallick BB, Kishore S, Das SK and Garg A (1985) Non-specific immunostimulation against viruses. *Comp Immunol Microbiol Infect Dis* **8**: 55–63

Miller T, Beausang LA, Meneghini M and Lidgard G (1993) Death-induced changes to the nuclear matrix: the use of anti-nuclear matrix antibodies to study agents of apoptosis. *Biotechniques* **15**: 1042–1047

Mizutani Y, Okada Y, Yoshida O, Fukumoto M and Bonavida B (1997) Doxorubicin sensitizes human bladder carcinoma cells to *Fas*-mediated cytotoxicity. *Cancer* **79**: 1180–1189

Morales A (1984) Long term results of BCG therapy for bladder cancer. *J Urol* **132**: 457–459

Morales A and Chin JL (1997) Mycobacterial cell wall (MCW) as an alternative to BCG in the treatment of carcinoma-in-situ (CIS) of bladder: an efficacy study. *J Urol* **157**: A214

 © Cancer Research Campaign 1999

Mosman T (1983) Rapid colorimetric assay for cellular growth and survival: application to proliferation and cytotoxicity assays. *J Immunol Methods* **65**: 55–63

Nastala CL, Edington HD, McKinney TG, Tahara H, Nalesnik MA, Brunda MJ, Gately MK, Wolf SF, Schreiber RD, Stewart WT, Storkus J and Lotze MT (1994) Recombinant interleukin-12 (IL-12) administration induces tumor regression in association with interferon-γ production. *J Immunol* **153**: 1697–1706

Newell MK, Haughn LJ, Maroun CR and Julius MH (1990) Death of mature T cells by separate ligation of CD4 and the T-cell receptor for antigen. *Nature* **347**: 286–288

Noguchi Y, Richards EC, Chen YT and Old LJ (1995) Influence of interleukin-12 on p53 peptide vaccination against established Meth A sarcoma. *Proc Natl Acad Sci USA* **92**: 2219–2223

Okamoto M, Hattori K and Oyasu R (1997) Interleukin-6 functions as an autocrine growth factor in human bladder carcinoma cell lines in vitro. *Int J Cancer* **72**: 149–154

Papapetropoulou M, Tsintzou A and Vantarakis A (1997) Environmental mycobacteria in bottled table waters in Greece. *Can J Microbiol* **43**: 499–502

Phillips NC, Gagné L, Ivanoff N and Riveau G (1996) Influence of phospholipid composition on antibody responses to liposome encapsulated protein and peptide antigens. *Vaccine* **14**: 898–904

Pryor K, Stricker P, Russell P, Golovsky D and Penny R (1995*a*) Antiproliferative effects of bacillus Calmette-Guérin and interferon α2b on human bladder cancer cells in vitro. *Cancer Immunol Immunother* **41**: 309–316

Pryor K, Goddard J, Goldstein D, Stricker P, Russell P, Govolsky D and Penny R (1995*b*) Bacillus Calmette-Guérin (BCG) enhances monocyte- and lymphocyte-mediated bladder tumour cell killing. *Br J Cancer* **71**: 801–807

Rahman M (1994) In vitro effects of high energy shock wave alone and combined with anticancer drugs on human bladder cancer cells. *Urol Int* **53**: 12–17

Rasheed S, Gardner MB, Rongey RW, Nelson-Rees A and Arnstein P (1977) Human bladder carcinoma: characterization of two new tumor cell lines and search for tumor viruses. *J Natl Cancer Inst* **58**: 881–887

Sasaki A, Kudoh S, Mori K, Takahashi N and Suzuki T (1997) Are BCG effects against urinary bladder carcinoma cell line T24 correlated with apoptosis in vitro? *Urol Int* **59**: 142–148

Shemtov MM, Cheng DL-W, Kong L, Shu W-P, Sassaroli M, Droller MJ and Liu BC-S (1995) LAK cell mediated apoptosis of human bladder cancer cells involves a pH-dependent endonuclease system in the cancer cell: possible mechanism of BGC therapy. *J Urol* **154**: 269–274

Stern AS, Magram J and Presky DH (1996) Interleukin-12 an integral cytokine in the immune response. *Life Sci* **58**: 639–654

Stine KC, Warren BA and Becton DL (1996) Apoptosis induced by interleukin-12 measured by DNA electrophoresis and in situ end labelling in leukemia. *Ann NY Acad Sci* **795**: 420–421

Tewari AK, Sharma NN, Rao JR, Mishra AK and Das SK (1996) Effect of *Mycobacterium phlei* on the development of immunity to *Babesia bigemina*. *Vet Parasitol* **62**: 223–230

Voest EE, Kenyon MN, O'Reilly MS, Truitt G, D'Amato RJ and Folkman J (1995) Inhibition of angiogenesis in vivo by interleukin 12. *J Natl Cancer Inst* **87**: 581–586

Wyllie SH (1980) Cell death: the significance of apoptosis. *Int Rev Cytol* **68**: 251–306

Zitvogel L and Lotze NH (1995) Role of interleukin-12 (IL-12) as an anti-tumor agent: experimental biology and clinical application. *Res Immunol* **146**: 628–638

Zlotta AR, Drowart A, Huygen K, De Bruyn J, Shekarsarai H, Decock M, Pirson M, Jurion F, Palfliet K, Denis O, Mascart F, Simon J, Schulman CC and Van Vooren JP (1997*a*) Humoral response against heat shock proteins and other mycobacterial antigens after intravesical treatment with bacille Calmette-Guérin (BCG) in patients with superficial bladder cancer. *Clin Exp Immunol* **109**: 157–165

Zlotta AR, Drowart A, Van Vorren JP, de Cook M, Pirson M, Palfliet K, Jurion F, Vanonckelen A, Simon J, Schulman CC and Huygen K (1997*b*) Evaluation and clinical significance of the T cell proliferative and cytokine response directed against the fibronectin binding antigen 85 complex of bacillus Calmette-Guérin during intravesical treatment of superficial bladder cancer. *J Urol* **157**: 492–498

Zou JP, Yamamoto N, Fujii T, Takenaka H, Kobayashi M, Herrmann SH, Wolf SF, Fujiwara H and Hamaoka T (1995) Systemic administration of rIL-12 induces complete tumor regression and protective immunity: response is correlated with a striking reversal of suppressed interferon-γ production by anti-tumor T cells. *Int Immunol* **7**: 1135–1145

*© Cancer Research Campaign 1999*
